# Supplementary material for: Learning curve and surgical outcome of robotic assisted colorectal surgery with ERAS program
Source: Sci Rep. 2022 Nov 29;12:20566. doi: 10.1038/s41598-022-24665-w (PMC9709162; doi:10.1038/s41598-022-24665-w)
Supplement: Supplementary file 1 — Supplementary Information 1. [file 41598_2022_24665_MOESM1_ESM.zip › LSC Raw data-Ñ[▒K/127/P_ñw╝╨░O▒Kñσ.pdf]

[redacted]

Pathologic diagnosis:
1. Ascending colon, terminal ileum, and appendix, lapa right hemicolectomy --- Diverticulitis with fecalith impaction and focal active serositis, of ascending colon. The appendix, ileocecal valve and bilateral cut ends are free.
2. IC root, resection --- Unremarkable fibrovascular tissue and lymph nodes (0/8).

Gross description:
The specimen consists of 1) a segment of colon, 9 cm in length, and 4.5 cm in proximal circumference, 3.5 cm in distal cut end circumference, and a segment of terminal ileum, 3.5 cm in length, and 2 cm in cut end circumference. An unremarkable appendix, 6.5x0.5 cm, is noted. There is an out-pouching lesion, 3x2.2 cm, noted in the ascneding colon, 3 cm to the nearest cut end. Some yellow fibrinoid exudate is noted on the serosa. On section, no mass lesion is identified grossly. The appendix, ileocecal valve, and both cut ends are free. 2) "IC root", a piece, 1.6x1.5x0.5 cm. Representative part for section: A) proximal cut end B) distal cut end C) ileocecal valve D) appendix E-H) lesion J) specimen 2.
#T-59420_2 #M-40630_2 0 1105 000000
#T-C4560_2 #M-09183_2 0 1105 000000
#T-59200_2 #M-09183_2 0 1105 000000

已電子簽章

敬告：本單僅供醫師參考使用，不具任何申請效力！！
請病患妥為保管，若不慎遺失需自負個人資料外洩責任！
